# Sputum Eosinophil and Macrophage Changes After Aspirin Challenge in Patients With Nonsteroidal Anti‐Inflammatory Drug–Exacerbated Respiratory Disease

**DOI:** 10.1002/clt2.70079

**Published:** 2025-09-10

**Authors:** Gabriela Trąd‐Wójcik, Piotr Szatkowski, Adam Ćmiel, Radosław Kacorzyk, Adam Stępień, Lucyna Mastalerz

**Affiliations:** ^1^ Second Department of Internal Medicine Jagiellonian University Medical College Krakow Poland; ^2^ Doctoral School of Medical and Health Sciences Jagiellonian University Krakow Poland; ^3^ Department of Applied Mathematics AGH University of Science and Technology Krakow Poland

**Keywords:** airway inflammatory phenotypes, aspirin challenge, aspirin hypersensitivity, induced sputum cells, sputum eosinophil percentage

## Abstract

**Background:**

Induced sputum cell count is crucial for assessing airway inflammatory phenotypes. This study investigated how aspirin‐induced bronchospasm affects sputum cell counts in patients with nonsteroidal anti‐inflammatory drug‐exacerbated respiratory disease (N‐ERD), comparing systemic versus local aspirin administration.

**Methods:**

Seventy‐eight patients with N‐ERD and 39 with aspirin‐tolerant asthma (ATA) participated. In the N‐ERD group, induced sputum was collected before aspirin challenge and during aspirin‐induced bronchospasm. We assessed changes in the percentages of eosinophils, neutrophils, lymphocytes, and macrophages, and airway inflammatory phenotypes classified by sputum cells into: (A) eosinophilic, neutrophilic, paucigranulocytic, and mixed; and (B) eosinophilic and noneosinophilic.

**Results:**

Baseline sputum neutrophil percentage was lower in the N‐ERD than in the ATA (32.9% ± 20.8% vs. 41.6% ± 22.5%; *p* = 0.02). Inflammatory phenotypes at baseline differed between groups in both classifications (A: *p* = 0.041; B: *p* = 0.044). In the N‐ERD group, sputum eosinophil percentage decreased after both oral (8.9% ± 11.6% vs. 6.1% ± 8.9%, *p* = 0.009) and inhaled (10.4% ± 16.1% vs. 4.8% ± 6.3%, *p* = 0.045) challenges, without altering inflammatory phenotypes. The ATA group showed no changes. Sputum macrophage percentage dropped after oral challenge in both groups (N‐ERD: 40.5% ± 18.5% vs. 35.6% ± 21.5%; *p* = 0.004; ATA: 36.5% ± 23.6% vs. 26.7% ± 20.4%; *p* = 0.0003). In the N‐ERD group, baseline sputum lymphocyte and eosinophil percentages were inversely correlated with the provocative dose of aspirin that resulted in a 20% decrease in baseline forced expiratory volume in 1 s following oral aspirin challenge (*R* = −0.31, *p* = 0.02 and *R* = −0.33, *p* = 0.02, respectively).

**Conclusion:**

In N‐ERD, sputum eosinophil percentage decreased after aspirin challenge regardless of administration route. In both N‐ERD and ATA, sputum macrophage percentage decreased after oral aspirin challenge.

## 1 Introduction

Nonsteroidal anti‐inflammatory drug (NSAID)‐exacerbated respiratory disease (N‐ERD) is a chronic condition characterized by eosinophilic and noneosinophilic inflammatory phenotypes of asthma based on induced sputum (IS) cells [1, 2], difficult to treat chronic rhinosinusitis with nasal polyps (CRwNPs), and acute respiratory reactions to aspirin and other cyclooxygenase‐1 (COX‐1) inhibitors [3, 4, 5]. CRSwNPs in N‐ERD patients is mainly associated with type 2 (T2) inflammation, although non‐T2 inflammation, including type 1 and type 3, can also occur [6]. N‐ERD involves several complex pathobiological mechanisms [3, 7, 8, 9]. While the disease has long been considered an eosinophilic inflammatory asthma [10], recent studies have shown that N‐ERD can present with either eosinophilic or noneosinophilic airway inflammation, with inflammatory phenotypes similar to those of asthma without aspirin hypersensitivity [1, 7, 11, 12, 13]. N‐ERD is classified as a type VII allergic reaction, characterized by a direct cellular and inflammatory response to chemical substances [14, 15].

The exact role of airway inflammatory cells in aspirin‐induced bronchospasm remains unclear, but eosinophils, mast cells, basophils, and other cells are thought to play a key role. Eosinophils, along with cysteinyl leukotrienes (cysLTs) and prostaglandin D_2_ (PGD_2_), are believed to significantly contribute to bronchospasm triggered by aspirin and other NSAIDs [3, 16]. Importantly, eosinophils, mast cells, basophils, epithelial cells, and Th2 lymphocytes can also produce these proinflammatory eicosanoids [3].

Previous studies showed that the sputum eosinophil count decreased during bronchospasm induced by inhaled aspirin in patients with N‐ERD [11]. However, the effect of oral aspirin challenge on individual sputum cell counts has not been sufficiently explored. In addition, no significant differences in airway inflammatory phenotypes based on induced sputum were observed following either oral or inhaled aspirin challenge tests in patients with N‐ERD and aspirin‐tolerant asthma (ATA) [11, 17].

Interestingly, a decrease in blood eosinophil count was noted after both types of aspirin challenge [3, 16, 18]. On the other hand, proinflammatory cysLT levels in induced sputum supernatant (ISS) increased after aspirin challenge tests [11, 17]. Urinary leukotriene E_4_ (LTE_4_) concentrations, an end product of systemic cysLT production, were elevated during aspirin‐induced reactions [3, 19]. However, no significant differences in PGD_2_ levels in ISS were found after either oral or bronchial challenges [11, 16, 18], although PGD_2_ levels in blood and urine increased after oral aspirin challenge [3, 16]. In contrast, anti‐inflammatory factors such as prostaglandin E_2_ and lipoxin A_4_ decreased in serum and locally in ISS after aspirin challenge [3, 11, 17, 20]. Recent evidence suggests that interactions between mast cells and epithelial cells can lead to the synthesis of 15‐oxo‐eicosatetraenoic acid (15‐oxo‐ETE) via hydroxyprostaglandin dehydrogenase (HPGD), an enzyme predominantly expressed in mast cells and localized near the nasal polyp epithelium of N‐ERD patients [21, 22, 23].

Current methods for assessing airway inflammatory phenotypes are not well established and typically rely on arbitrary cut‐off values for sputum eosinophil and neutrophil counts. These values vary according to the authors' preferences and differ between studies [1, 11, 18, 24, 25]. A 2% cut‐off for sputum eosinophilia is used in severe asthma for T2 phenotyping [26], but it remains unclear whether evaluating individual sputum cell counts (including eosinophils, neutrophils, lymphocytes, and macrophages) would provide more accurate insights. Furthermore, no studies have compared the effects of oral and inhaled aspirin challenges on sputum cell counts in patients with N‐ERD versus those with ATA.

The aim of this study was to assess how aspirin‐induced bronchospasm affect sputum cell percentages and airway inflammatory phenotypes in N‐ERD patients, comparing the effects of systemic (oral) and local (inhaled) aspirin delivery. Another aim was to evaluate whether measuring individual sputum cell percentages or identifying overall inflammatory patterns provides a better understanding of how aspirin impacts airway inflammation.

## 2 Methods

### 2.1 Patients

A total of 117 patients were enrolled in the study, including 78 patients with N‐ERD and 39 patients with ATA as controls. The aspirin challenge test was performed either orally or via inhalation, as previously described [27].

In the N‐ERD group, 52 patients underwent oral aspirin challenge, while 26 patients underwent inhaled aspirin challenge. In the ATA group, 23 patients underwent oral aspirin challenge, and 16 patients underwent inhaled aspirin challenge. Before the study, patients were treated only with inhaled corticosteroids, long‐acting β_2_‐agonists, nasal corticosteroids, and antihistamines. Treatment with long‐acting β_2_‐agonists was discontinued prior to the aspirin challenge, as per the European Academy of Allergy and Clinical Immunology guidelines [27]. Antihistamines were discontinued 6 weeks before the study. None of the patients had used antileukotrienes, biologics, or oral corticosteroids in the past 6 months. In addition, all patients had stable asthma without pulmonary tract infections or exacerbations in the past 6 months. The ATA cohort had not taken aspirin or other NSAIDs for 6 weeks before the aspirin challenge. On the day of the challenge, each patient had a baseline forced expiratory volume in 1 s (FEV_1_) of 70% or higher. The characteristics of the study groups are shown in Table 1.

**TABLE 1:** Characteristics of the study groups.

Variable	All study participants			Oral aspirin challenge			Inhaled aspirin challenge		
	N‐ERD (*n* = 78)	ATA (*n* = 39)	*p* value	N‐ERD (*n* = 52)	ATA (*n* = 23)	*p* value	N‐ERD (*n* = 26)	ATA (*n* = 16)	*p* value
Age, years	45.9 ± 11.6	48.0 ± 11.0	0.370	48.9 ± 11.5	47.4 ± 8.9	0.596	40.0 ± 9.6	48.9 ± 15.5	0.256
Male sex, *n* (%)	20 (25.7)	15 (38.5)	0.153	13 (25.0)	8 (34.8)	0.384	7 (26.9)	7 (43.8)	0.261
BMI (kg/m^2^)	27.5 ± 9.8	26.6 ± 4.1	0.592	26.5 ± 4.8	26.6 ± 3.9	0.466	29.5 ± 15.7	26.5 ± 4.4	0.889
Asthma duration, years	11.8 ± 8.7	12.2 ± 10.0	0.913	12.9 ± 9.1	11.8 ± 11.2	0.642	10.0 ± 7.6	12.8 ± 7.9	0.205
ACT score	22 ± 3	23 ± 3	0.125	22 ± 3	22 ± 3	0.725	22 ± 3	24 ± 2	0.065
Asthma control based on the ACT score, *n* (%)			0.680			0.896			0.411
Well controlled	63 (80.8)	33 (86.8)		41 (78.8)	19 (82.6)		22 (84.6)	14 (87.5)	
Partially controlled	13 (16.7)	4 (10.5)		9 (17.3)	3 (13.1)		4 (15.4)	2 (12.5)	
Uncontrolled	2 (2.6)	1 (2.6)		2 (3.9)	1 (4.3)		0	0	
Asthma severity, *n* (%)			0.081			0.492			0.097
Mild	8 (10.3)	10 (26.3)		7 (13.4)	5 (21.8)		2 (7.7)	5 (33.33)	
Moderate	40 (51.3)	16 (42.1)		29 (55.8)	11 (47.8)		10 (38.5)	5 (33.33)	
Severe	30 (38.5)	12 (31.6)		16 (30.8)	7 (30.4)		14 (53.8)	5 (33.33)	
ICS dose (fluticasone propionate or equivalent), μg	500 (400–1000)	500 (400–1000)	0.513	500 (400–1000)	450 (250–1000)	0.475	1000 (400–1000)	580 (400–1000)	0.582
Baseline FEV_1_ (% predicted)	92.8 ± 14.5	100.5 ± 15.9	**0.011**	92.7 ± 14.4	100.7 ± 16.0	**0.036**	93.1 ± 14.2	100.1 ± 16.2	0.155
CRSwNPs, *n* (%) (yes)	78 (100.0)	32 (82.1)	**<** **0.001**	52 (100.0)	21 (91.3)	**0.034**	26 (100.0)	11 (68.8)	**0.002**
Peripheral blood eosinophil count, cells/μL	470.7 ± 332.6	264.8 ± 160.8	**<** **0.001**	472.2 ± 362.9	302.6 ± 141.0	**0.039**	467.8 ± 272.9	210.5 ± 176.1	**0.001**
IgE, IU/mL	914.0 ± 619.8	336.3 ± 118.9	0.148	201.3 ± 290.0	182.9 ± 365.8	0.653	208.3 ± 295.9	256.8 ± 303.1	0.739
Positive skin prick test, *n* (%)	26 (33.3)	18 (46.2)	0.178	16 (30.8)	10 (43.5)	0.286	10 (38.5)	8 (50.0)	0.463
SNOT‐22	44 (35–59)	29 (22–38)	**0.002**	50 (39–62)	34 (29–40)	**0.002**	36 (29–41)	20 (15–23)	**0.026**
Lund‐Mackay score	18 (13–20)	11 (9–14)	**0.001**	15 (13–20)	14 (11–14)	**0.036**	18 (13–21)	7 (3–10)	**0.005**

*Note:* Continuous data are presented as mean ± standard deviation or median (interquartile range) unless otherwise indicated. Bold values indicate statistically significant differences (*p* < 0.05).

Abbreviations: ACT, Asthma Control Test; ATA, aspirin‐tolerant asthma; BMI, body mass index; CRSwNPs, chronic rhinosinusitis with nasal polyps; FEV_1_, forced expiratory volume in 1 s; ICS, inhaled corticosteroid; IgE, immunoglobulin E; N‐ERD: nonsteroidal anti‐inflammatory drug‐exacerbated respiratory disease; SNOT‐22, 22‐item Sino‐Nasal Outcome Test.

The study was approved by the Jagiellonian University Ethics Committee, and all participants provided written informed consent. The study was conducted in accordance with the Declaration of Helsinki.

### 2.2 Study Design

Six weeks before the study, patients visited the outpatient clinic for treatment evaluation and skin prick tests (if they had not been performed previously). After this run‐in period, a 3‐day hospitalization was scheduled. In patients with aspirin hypersensitivity, a 20% decrease in FEV_1_ was observed during the aspirin challenge, and the provocative dose (PD20) required to cause this reduction was calculated. Oral aspirin challenge was performed on 2 days, while the inhaled challenge was completed in 1 day [27]. Sputum samples were collected twice: initially before placebo in the oral challenge or upon admission in the inhaled challenge, and during the acute hypersensitivity reaction in patients with N‐ERD. In the ATA group, samples were collected 1.5 h (oral) or 30 min (inhaled) after the last dose of aspirin, when the cumulative dose was reached, and a negative test result was obtained.

**FIGURE 1:**
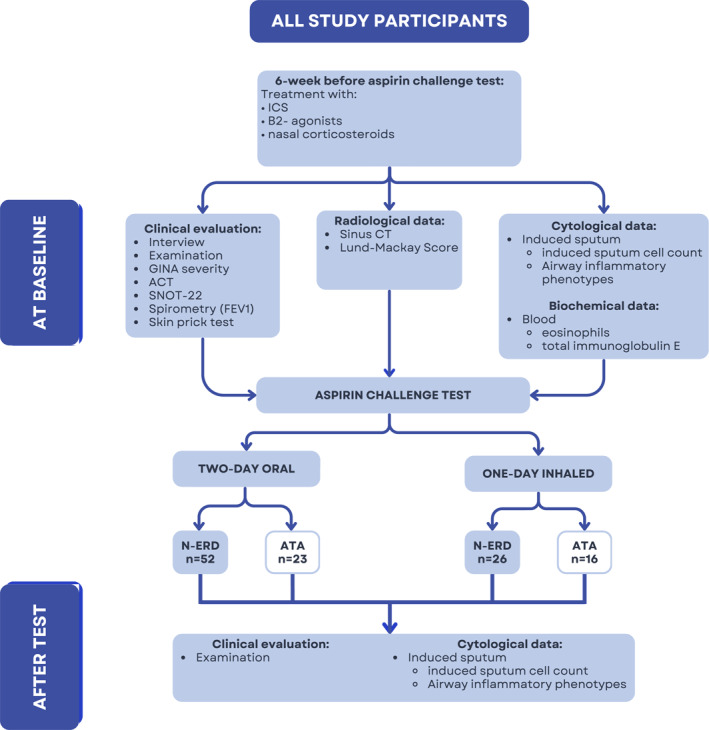
The flow of the study. ACT, Asthma Control Test; ATA, aspirin tolerant asthma; CT, computed tomography; FEV1, forced expiratory volume in the first second; GINA, Global Initiative for Asthma; ICS, inhaled corticosteroids; N‐ERD, non‐steroidal anti‐inflammatory drug‐exacerbated respiratory disease; SNOT‐22, the 22‐item Sino‐Nasal Outcome Test.

Demographic, clinical, and biochemical data, computed tomography scans of the nasal sinuses, and spirometry results were assessed for all participants. Asthma control was evaluated using the Asthma Control Test (ACT) [26], and severity was assessed according to the GINA guidelines [26]. Nasal symptoms were evaluated using the 22‐item Sino‐Nasal Outcome Test (SNOT‐22) [28]. Two radiologists assessed sinus radiological changes using the Lund‐Mackay score [29]. The study design is illustrated in Figure 1.

### 2.3 Induced Sputum

Induced sputum was collected according to the European Respiratory Society recommendations before and after aspirin challenge [30]. The procedure was described in detail previously [11, 12, 17]. Sputum cell counts were expressed as a percentage of 800 sputum cells (of squamous and ciliated epithelia, eosinophils, neutrophils, macrophages, and lymphocytes) [31].

### 2.4 Sputum Cell Percentage Classifications

Sputum cells were classified and assessed according to the following 2 categories:

1. sputum cell counts including eosinophils, neutrophils, lymphocytes, and macrophages. The cell counts were expressed as a percentage of 800 cells, which included squamous and ciliated epithelial cells, eosinophils, neutrophils, macrophages, and lymphocytes [31, 32, 33, 34].
2. airway inflammatory phenotypes based on sputum cells, classified into:
  - eosinophilic (≥ 3% eosinophils and < 64% neutrophils), neutrophilic (≥ 64% neutrophils and < 3% eosinophils), mixed (≥ 3%, eosinophils and ≥ 64% neutrophils), and paucigranulocytic (< 3% eosinophils and < 64% neutrophils) [1, 17, 24].
  - eosinophilic (eosinophils ≥ 3%) and noneosinophilic (eosinophils < 3%).

In this classification, the mixed phenotype was considered a transitional state between eosinophilic and neutrophilic phenotypes and was not included [25].

### 2.5 Statistical Analysis

Details are provided in Supporting Information S1.

## 3 Results

*Comparison of N‐ERD (n = 78) and ATA (n = 39) groups before oral or inhaled aspirin challenge.*

There were no differences in age, sex, body mass index, asthma control, asthma severity, inhaled corticosteroid dose, asthma duration, serum immunoglobulin E levels, and rates of positive skin prick test results between patients with N‐ERD and ATA. CRSwNPs was more common in patients with N‐ERD than in those with ATA (100% vs. 82.1%, *p* < 0.001). The mean baseline FEV_1_ was lower in the N‐ERD than in the ATA group (92.8 ± 14.5 mL vs. 100.5 ± 15.9 mL, *p* = 0.011). Conversely, the mean blood eosinophil count was higher in the N‐ERD group than in the ATA group (470.7 ± 332.6/mm^3^ vs. 264.8 ± 160.8/mm^3^, *p* < 0.001). The median Lund‐Mackay and SNOT‐22 scores were higher in patients with N‐ERD than in those with ATA (18 [IQR, 13–20] vs. 11 [IQR, 9–14], *p* = 0.001 and 44 [IQR, 35–59] vs. 29 [IQR, 22–38], *p* = 0.002, respectively). Detailed characteristics of the study groups are shown in Table 1.

*Comparison of N‐ERD (n = 52) and ATA (n = 23) groups before oral aspirin challenge*.

Details are provided in Supporting Information S1.

*Comparison of N‐ERD (n = 26) and ATA (n = 16) groups before inhaled aspirin challenge*.

Details are provided in Supporting Information S1.

*Sputum eosinophil percentage at baseline and after oral or inhaled aspirin challenge in the N‐ERD (n = 78) and ATA (n = 39) groups*.

**FIGURE 2:**
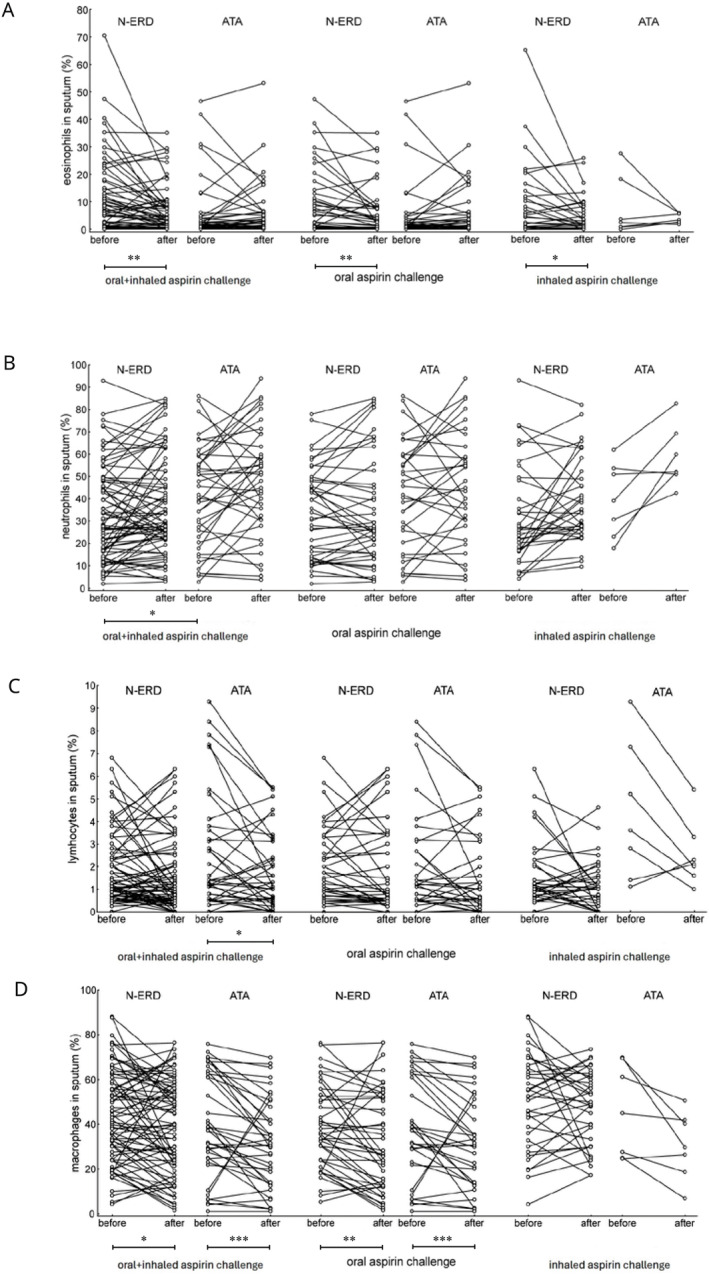
Induced sputum cell counts before and after aspirin challenge test: (A) eosinophil cell percentage; (B) neutrophil cell percentage; (C) lymphocyte cell percentage; (D) macrophage cell percentage. * *p* value < 0.05; ** *p* value < 0.01; *** *p* value < 0.001. ATA, aspirin‐tolerant asthma; N‐ERD, non‐steroidal anti‐inflammatory drug‐exacerbated respiratory disease.

There were no differences in baseline eosinophil percentage between the N‐ERD and ATA groups. After the aspirin challenge, patients with N‐ERD showed a 1.68‐fold reduction in the mean eosinophil percentage (9.4% ± 13.2% vs. 5.6% ± 8.1%, *p* = 0.001), while no changes were observed in the ATA group (Figure 2A).

*Sputum eosinophil count percentage at baseline and after oral aspirin challenge in the N‐ERD (n = 52) and ATA (n = 23) groups*.

At baseline, there were no differences between the N‐ERD and ATA groups. After the oral challenge, patients with N‐ERD had a 1.46‐fold reduction in the mean sputum eosinophil percentage (8.9% ± 11.6% vs. 6.1 ± 8.9%, *p* = 0.009), with no changes in ATA (Figure 2A).

*Sputum eosinophil percentage at baseline and after inhaled aspirin challenge in the N‐ERD (n = 26) and ATA (n = 16) groups*.

At baseline, there were no differences between groups. After inhaled aspirin challenge, patients with N‐ERD had a 2.17‐fold reduction in the mean sputum eosinophil percentage (10.4% ± 16.1% vs. 4.8% ± 6.3%, *p* = 0.045), while no changes were observed in the ATA group (Figure 2A).

*Sputum neutrophil percentage at baseline and after oral or inhaled aspirin challenge in the N‐ERD (n = 78) and ATA (n = 39) groups*.

At baseline, the mean sputum neutrophil percentage was lower in patients with N‐ERD than in those with ATA (32.9% ± 20.8% vs. 41.6% ± 22.5%, *p* = 0.039). No significant changes were observed after oral aspirin challenge in either group (Figure 2B).

*Sputum neutrophil percentage at baseline and after oral aspirin challenge in the N‐ERD (n = 52) and ATA (n = 23) groups*.

Details are provided in Supporting Information S1.

*Sputum neutrophil percentage at baseline and after inhaled aspirin challenge in the N‐ERD (n = 26) and ATA (n = 16) groups*.

Details are provided in Supporting Information S1.

*Sputum lymphocyte percentage at baseline and after oral or inhaled aspirin challenge in the N‐ERD (n = 78) and ATA (n = 39) groups*.

At baseline, there were no differences in sputum lymphocyte percentage between N‐ERD and ATA. After the aspirin challenge, there was a 1.37‐fold reduction in the mean lymphocyte percentage in patients with ATA (2.6% ± 2.5% vs. 1.9% ± 1.7%, *p* = 0.029), while no significant differences were observed in patients with N‐ERD (Figure 2C).

*Sputum lymphocyte percentage at baseline and after oral aspirin challenge in the N‐ERD (n = 52) and ATA (n = 23) groups*.

Details are provided in Supporting Information S1.

*Sputum lymphocyte percentage at baseline and after inhaled aspirin challenge in the N‐ERD (n = 26) and ATA (n = 16) groups*.

Details are provided in Supporting Information S1.

*Sputum macrophage percentage at baseline and after oral or inhaled aspirin challenge in the N‐ERD (n = 78) and ATA (n = 39) groups*.

At baseline, there were no differences in sputum macrophage percentage between the N‐ERD and ATA groups. After the aspirin challenge, there was a 1.12‐fold reduction in the mean macrophage percentage in the N‐ERD group (43.9% ± 20.6% vs. 39.1% ± 20.8%, *p* = 0.014) and a 1.28‐fold reduction in the ATA group (39.7% ± 22.9% vs. 31.1% ± 19.8%; *p* < 0.001) (Figure 2D).

*Sputum macrophage percentage at baseline and after oral aspirin challenge in the N‐ERD (n = 52) and ATA (n = 23) groups*.

At baseline, there were no differences in sputum macrophage percentage between the N‐ERD and ATA groups. After oral aspirin challenge, there was a 1.14‐fold reduction in the mean sputum macrophage percentage in the N‐ERD group (40.5% ± 18.5% vs. 35.6% ± 21.5%, *p* = 0.004) and a 1.37‐fold reduction in the ATA group (36.5% ± 23.6% vs. 26.7% ± 20.4%, *p* < 0.001) (Figure 2D).

*Sputum macrophage percentage at baseline and after inhaled aspirin challenge in the N‐ERD (n = 26) and ATA (n = 16) groups*.

At baseline, there were no differences in sputum macrophage percentage between the N‐ERD and ATA groups. In addition, no significant changes in sputum macrophage percentage were noted after inhaled aspirin challenge in either group (Figure 2D).

*Differences in eosinophilic, neutrophilic, paucigranulocytic, and mixed airway inflammatory phenotypes between the ATA and N‐ERD groups*.

The analysis of all patients revealed significant differences in inflammatory phenotypes between the N‐ERD and ATA groups at baseline (*p* = 0.041). The eosinophilic phenotype was more common in patients with N‐ERD than in those with ATA (50% vs. 28.2%), while paucigranulocytic and neutrophilic phenotypes were less common in patients with N‐ERD than in those with ATA (paucigranulocytic: 41% vs. 56.4%; neutrophilic: 9% vs.10.3%). The mixed phenotype was observed in 5.1% of the patients with ATA, and in none of the patients with N‐ERD. Due to small sample sizes, the statistical analysis of differences after aspirin challenge was not feasible.

In the analysis based on the type of aspirin challenge (oral vs. inhaled), there were no differences between the N‐ERD and ATA groups at baseline. Due to small sample sizes, the statistical analysis of differences after aspirin challenge was not feasible.

**FIGURE 3:**
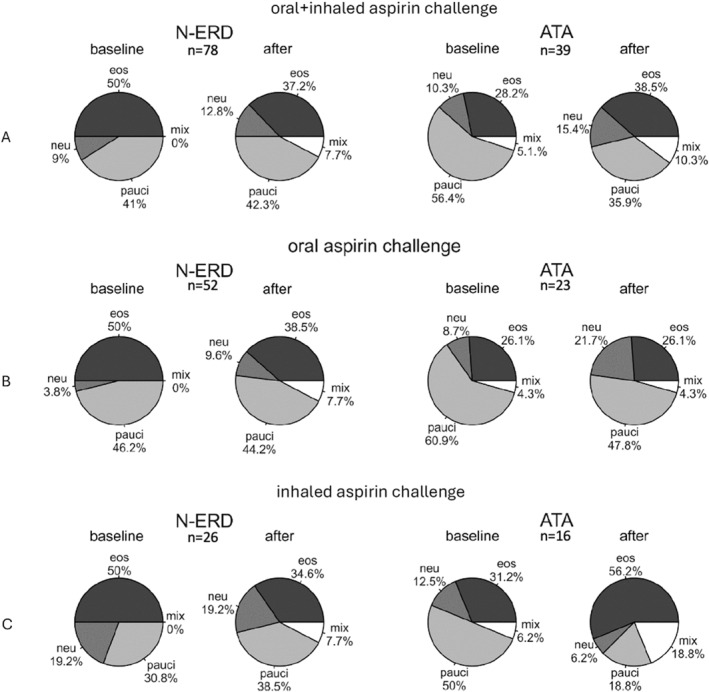
Changes in the rates of eosinophilic, neutrophilic, paucigranulocytic, and mixed airway inflammatory phenotypes before and after aspirin challenge. (A) before and after oral and inhaled aspirin challenge test; (B) before and after oral aspirin challenge test; (C) before and after inhaled aspirin challenge test. ATA, aspirin‐tolerant asthma; eos, eosinophilic; mix, mixed; N‐ERD, nonsteroidal anti‐inflammatory drug‐exacerbated respiratory disease; neu, neutrophilic; pauci, paucigranulocytic.

The rates of airway inflammatory phenotypes before and after aspirin challenge are presented in Figure 3. Changes in the phenotypes before and after aspirin challenge are presented in Table 2.

**TABLE 2:** A—Changes in eosinophilic, neutrophilic, paucigranulocytic and mixed airway inflammatory phenotypes before and after aspirin challenge test; B—changes in eosinophilic and noneosinophilic airway inflammatory phenotypes before and after aspirin challenge test.

A	Phenotype at baseline	Amount	Eos after challenge	Neu after challenge	Pauci after challenge	Mixed after challenge
N‐ERD						
All study participants						
Number	Eos	39	26	0	7	6
% of changes			67	0	18	15
Number	Neu	7	0	6	1	0
% of changes			0	86	14	0
Number	Pauci	32	3	4	25	0
% of changes			9	13	78	0
Number	Mixed	0	0	0	0	0
% of changes			0	0	0	0
Oral aspirin challenge test						
Number	Eos	26	18	0	4	4
% of changes			70	0	15	15
Number	Neu	2	0	2	0	0
% of changes			0	100	0	0
Number	Pauci	24	2	3	19	0
% of changes			8	13	79	0
Number	Mixed	0	0	0	0	0
% of changes			0	0	0	0
Inhaled aspirin challenge test						
Number	Eos	13	8	0	3	2
% of changes			62	0	23	15
Number	Neu	5	0	4	1	0
% of changes			0	80	20	0
Number	Pauci	8	1	1	6	0
% of changes			13	13	75	0
Number	Mixed	0	0	0	0	0
% of changes			0	0	0	0
ATA						
All study participants						
Number	Eos	11	9	1	0	1
% of changes			82	9	0	9
Number	Neu	4	0	2	1	1
% of changes			0	50	25	25
Number	Pauci	22	5	3	13	1
% of changes			23	14	59	5
Number	Mixed	2	1	0	0	1
% of changes			50	0	0	50
Oral aspirin challenge test						
Number	Eos	6	4	1	0	1
% of changes			67	17	0	17
Number	Neu	2	0	2	0	0
% of changes			0	100	0	0
Number	Pauci	14	1	2	11	0
% of changes			7	14	79	0
Number	Mixed	1	1	0	0	0
% of changes			100	0	0	0
Inhaled aspirin challenge test						
Number	Eos	5	5	0	0	0
% of changes			100	0	0	0
Number	Neu	2	0	0	1	1
% of changes			0	0	50	50
Number	Pauci	8	4	1	2	1
% of changes			50	13	25	13
Number	Mixed	1	0	0	0	1
% of changes			0	0	0	100

B	Phenotype at baseline	Amount	Eos after challenge	Non‐eos after challenge	Mixed^a^ after challenge
N‐ERD					
All study participants					
Number	Eos	39	26	8	5
% of changes			67	21	13
Number	Non‐eos	39	3	36	0
% of changes			8	92	0
Number	Mixed^a^	0	0	0	0
% of changes			0	0	0
Oral aspirin challenge test					
Number	Eos	26	18	4	4
% of changes			69	15	15
Number	Non‐eos	26	2	24	0
% of changes			8	92	0
Number	Mixed^a^	0	0	0	0
% of changes			0	0	0
Inhaled aspirin challenge test					
Number	Eos	13	8	4	1
% of changes			62	31	8
Number	Non‐eos	13	1	12	0
% of changes			8	92	0
Number	Mixed^a^	0	0	0	0
% of changes			0	0	0
ATA					
All study participants					
Number	Eos	11	9	1	1
% of changes			82	9	9
Number	Non‐eos	26	5	19	2
% of changes			19	73	8
Number	Mixed^a^	2	1	0	1
% of changes			50	0	50
Oral aspirin challenge test					
Number	Eos	6	4	1	1
% of changes			67	17	17
Number	Non‐eos	16	1	15	0
% of changes			6	94	0
Number	Mixed^a^	1	1	0	0
% of changes			100	0	0
Inhaled aspirin challenge test					
Number	Eos	5	5	0	0
% of changes			100	0	0
Number	Non‐eos	10	4	4	2
% of changes			40	40	20
Number	Mixed^a^	1	0	0	1
% of changes			0	0	100

Abbreviations: ATA, aspirin‐tolerant asthma; Eos, eosinophilic; N‐ERD, nonsteroidal anti‐inflammatory drug‐exacerbated respiratory disease; Neu, neutrophilic; Non‐eos, non‐eosinophilic; Pauci, paucigranulocytic.

^a^ The mixed phenotype is not included in the classification.

*Eosinophilic and noneosinophilic airway inflammatory phenotypes*.

**FIGURE 4:**
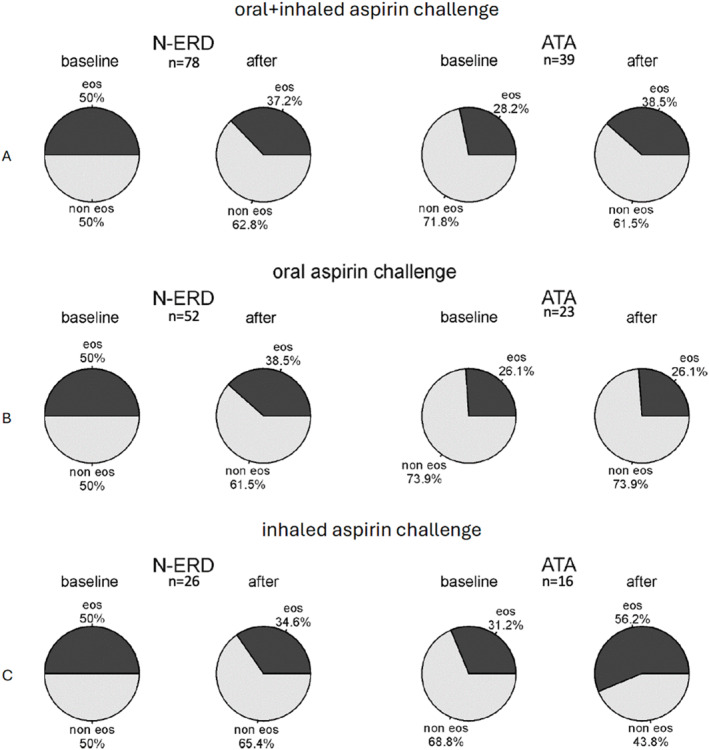
Changes in the rates of airway inflammatory phenotypes before and after aspirin challenge test based on 2 airway inflammatory subphenotypes (eosinophilic and noneosinophilic). (A) before and after oral and inhaled aspirin challenge test; (B) before and after oral aspirin challenge test; (C) before and after inhaled aspirin challenge test. ATA, aspirin‐tolerant asthma; eos, eosionophilic; N‐ERD, nonsteroidal anti‐inflammatory drug‐exacerbated respiratory disease; non‐eos, noneosinophilic.

The analysis of all patients revealed significant differences in inflammatory phenotypes between the N‐ERD and ATA groups at baseline (*p* = 0.044). The eosinophilic phenotype was more common in patients with N‐ERD than in those with ATA (50% vs. 28.2%). After the aspirin challenge, the rate of the eosinophilic phenotype decreased from 50% to 37.2% in the N‐ERD group (*p* = 0.021), while there was a nonsignificant increase in the ATA group (Figure 4A).

In the analysis based on the type of aspirin challenge (oral vs. inhaled), there were no differences between the N‐ERD and ATA groups at baseline and after aspirin challenge.

The rates of airway inflammatory phenotypes before and after aspirin challenge are presented in Figure 4. Changes in inflammatory phenotypes before and after aspirin challenge are shown in Table 2.

*Correlations between sputum cell percentage, airway inflammatory phenotypes, and PD20*.

In patients with N‐ERD undergoing oral challenge, there was a significant negative correlation between baseline sputum lymphocyte percentage and PD20 (*R* = −0.31, *p* = 0.025), as well as between baseline sputum eosinophil percentage and PD20 (*R* = −0.33, *p* = 0.017). No significant correlations were found between airway inflammatory phenotypes and PD20 before or after aspirin challenge.

*Correlations between blood eosinophils and sputum inflammatory cells*.

In the N‐ERD group, there was a positive correlation between blood eosinophil count and both eosinophil sputum percentage (*R* = 0.26, *p* = 0.024), and sputum lymphocyte percentage (*R* = 0.31, *p* = 0.008). No correlations were observed in the ATA group.

*Correlation between sputum eosinophil percentage and uLTE*_*4*_.

There was a positive correlation between sputum eosinophil percentage in the N‐ERD group (*R* = 0.559, *p* < 0.001) and in the ATA group (*R* = 0.349, *p* = 0.034).

## 4 Discussion

This study is the first to compare changes in sputum cell counts and airway inflammatory phenotypes based on sputum cells after systemic or local aspirin administration in patients with N‐ERD and ATA. We evaluated whether detailed sputum cell analysis [31, 32, 33, 34] or traditional airway inflammatory phenotype classification [24, 25] better explains the pathogenesis of aspirin‐induced bronchospasm in N‐ERD.

Aspirin‐induced bronchospasm, regardless of the route of aspirin administration (oral or inhaled), was associated with reduced eosinophil counts in both blood and sputum in patients with N‐ERD. One possible explanation is that eosinophils become activated and retained in the airway tissue during aspirin‐induced reactions, leading to reduced counts in blood and sputum. Szczeklik et al. [35] demonstrated increased eosinophil numbers in bronchoalveolar lavage fluid (BAL) after bronchial aspirin challenge without a corresponding rise in eosinophil cationic protein (ECP), suggesting tissue sequestration rather than degranulation. In contrast, elevated ECP levels in nasal lavage after aspirin challenge indicate eosinophilic activation in the upper airway, consistent with findings in nasal polyps from N‐ERD patients [36, 37]. Laidlaw and Boyce [38] provide a comprehensive overview of the complex network of mediator and cellular interactions driving eosinophils recruitment and activation in N‐ERD, highlighting its distinct pathophysiology compares to classical eosinophilic asthma. This study showed that a decrease in blood eosinophil count after aspirin challenge can indicate eosinophil recruitment to tissues.

Although our study did not include airway tissue or BALF sampling, the observed changes in blood and sputum eosinophil count may reflect tissue‐level activation. Further studies using bronchial biopsies or BALF during aspirin‐induced bronchospasm are needed to better understand eosinophil dynamics in N‐ERD.

Interestingly, during chronic aspirin treatment after aspirin desensitization, blood eosinophil counts increased [16, 18], while sputum eosinophil percentage decreased [18, 39] in patients with N‐ERD. This suggests reduced eosinophil recruitment into lung tissue, potentially limiting their inflammatory role.

Eosinophilic inflammation is common in asthma but is not specific to N‐ERD. Interleukin IL‐5 regulates eosinophil recruitment and activation; however, its blood levels are not elevated in N‐ERD [40]. This implies that eosinophils, despite low IL‐5 levels, can still become activated in the lung tissue during aspirin‐induced reactions. Eosinophil activation may result in their migration into lung tissue, resulting in reduced levels in blood and sputum, which could contribute to aspirin‐induced bronchospasm.

In allergic conditions, eosinophil progenitor cells are upregulated in various tissues (such as blood, bone marrow, and lung), showing enhanced ability to grow and migrate in response to stimuli [41]. A similar phenomenon might occur during aspirin‐induced bronchospasm. Therefore, studying eosinophils in bronchial biopsies could enhance our understanding of their role in N‐ERD. Eosinophils have additional properties, such as consistent expression of leukotriene C_4_ synthase, as well as the production of interferon‐γ and PGD_2_ [42], which stimulate the release of urinary LTE_4_ during acute aspirin reactions [19]. In contrast to ATA patients, aspirin induces bronchospasm in N‐ERD patients, complicating the differentiation between changes in sputum cells due to COX‐1 inhibition and those due to bronchospasm trauma.

Thus, our findings highlight the need to assess both systemic and local eosinophil activity in aspirin‐induced asthma. It would be interesting to examine the airway inflammatory phenotype simultaneously using samples from the bronchial mucosa. If the phenotype does not change, it would suggest that eosinophils are stored in lung tissue. The conventional classification of airway inflammatory phenotypes did not capture the dynamic changes in sputum cells during bronchospasm in our study. This indicates that detailed analyses of specific cells, such as lymphocytes, eosinophils, neutrophils, and macrophages, might provide better insights than traditional phenotype categorization.

An interesting finding from our study is that aspirin reduced sputum macrophage percentage, regardless of the occurrence of bronchospasm. This reduction was consistent in both N‐ERD and ATA patients, suggesting that aspirin's anti‐inflammatory effects may suppress macrophage activation and migration into the airways. It is known that N‐ERD patients have higher concentrations of mast cells in the nasal and bronchial mucosa compared to ATA patients [3, 20]. In addition, macrophages in patients with N‐ERD undergo metabolic and epigenetic changes, leading to a persistent proinflammatory state. These macrophages produce higher levels of acylcarnitine, proinflammatory arachidonic acid metabolites, cytokines, and chemokines compared to healthy individuals, which intensifies inflammation and airway hyperreactivity [43, 44, 45]. This chronic macrophage activation likely contributes to therapy‐resistant airway inflammation observed in N‐ERD [46]. It is important to note that a paucigranulocytic inflammatory phenotype in N‐ERD is often associated with macrophage infiltration, highlighting the role of these immune cells in chronic airway inflammation [47]. Recent research has highlighted the role of 15‐oxo‐ETE, a metabolite produced by macrophages, which may play a significant role in the pathogenesis of N‐ERD [21, 23, 48, 49]. Furthermore, we observed that higher percentages of eosinophils and lymphocytes in IS correlate with a lower dose of orally administered aspirin required to cause a 20% decrease in FEV_1_ in N‐ERD patients. This suggests that patients with elevated sputum eosinophil and lymphocyte percentages may have heightened airway hypersensitivity to aspirin. Interestingly, this correlation was not observed when aspirin was administered via inhalation, which may be related to the different pharmacodynamics of aspirin depending on the route of administration [27]. Although baseline sputum eosinophil and lymphocyte percentage did not differ significantly between N‐ERD and ATA groups, the negative correlation within N‐ERD cohort suggests that individual airway inflammation levels better predict aspirin sensitivity than group averages. There is pronounced accumulation and activation of eosinophils within airway tissues and IS, which contribute to inflammation and bronchial hyperresponsiveness in N‐ERD. Activated eosinophils release proinflammatory mediators, including cysteinyl leukotrienes, ECP, and eosinophil peroxidase (EPX), all of which contribute to bronchoconstriction, epithelial damage and airway remodeling [16, 38, 50, 51]. Our study measured only total sputum lymphocyte percentages without subpopulation analysis, limiting interpretation. Emerging evidence shows that lymphocyte subsets like ILC2s and Th2 cells secrete cytokines (IL‐4, IL‐5, IL‐13) driving eosinophil activation. Cytokines such as IL‐9 and IL‐25 also promote mast cell activation and mucus production, worsening bronchoconstriction [52, 53, 54]. It remains unclear why different doses of aspirin, whether systemic or local, elicit similar hypersensitivity reactions in N‐ERD, regardless of the airway inflammatory phenotype. Our findings emphasize the need for a closer examination of the number and phenotypes of lymphocytes in the airway infiltrate, including both innate and adaptive immune lymphocytes, to determine whether T2 or non‐T2 inflammation predominates in N‐ERD.

Our study has several limitations. First, comparing the effects of aspirin challenge between asthma patients with and without aspirin hypersensitivity introduces potential uncertainty as to the findings. Variability in the cumulative doses of aspirin administered to patients with positive versus negative aspirin challenge results could impact the outcomes. Additionally, aspirin‐induced bronchospasm was observed only in patients with aspirin hypersensitivity, so caution is warranted when interpreting comparisons with the ATA group. Daily fluctuations in blood eosinophil counts may also influence the results [55]. Finally, it remains unclear whether the time of day affects sputum eosinophil percentage, as some studies reported no significant changes [56], while others suggested variability based on the time of day [55].

## 5 Conclusion

In summary, aspirin‐induced bronchospasm leads to a reduction in sputum eosinophil percentage only in patients with N‐ERD. Moreover, oral aspirin challenge reduces sputum macrophage percentage both in asthmatics with aspirin hypersensitivity and those with ATA. Both oral and inhaled aspirin challenge do not alter airway inflammatory phenotypes based on sputum cells in asthmatics, regardless of aspirin hypersensitivity.

Higher sputum eosinophil and lymphocyte percentages at baseline correlate with a lower oral provocative dose of aspirin needed to induce bronchospasm. Future studies should investigate lymphocyte phenotyping in blood, sputum, bronchial mucosa, and BAL before and during aspirin‐induced bronchospasm.

Our findings suggest that traditional inflammatory phenotype classifications, while somewhat “artificial,” still hold value in clinical settings for guiding biological treatment of asthma. However, evaluating the detailed cell composition in sputum samples may provide deeper insights in research contexts.

## Author Contributions

**Gabriela Trad‐Wojcik:** writing – original draft, investigation, validation, visualization, software, formal analysis, data curation, methodology. **Piotr Szatkowski:** methodology, software, writing – review and editing, formal analysis, visualization, data curation. **Adam Ćmiel:** software, writing – original draft, visualization, formal analysis, data curation. **Radosław Kacorzyk:** writing – review and editing, methodology, validation, software, formal analysis, investigation. **Adam Stępień:** writing – review and editing, methodology, software. **Lucyna Mastalerz:** conceptualization, funding acquisition, writing – original draft, validation, supervision, investigation, methodology, visualization, software, formal analysis, project administration, data curation, resources.

## Conflicts of Interest

The authors declare no conflicts of interest.

## Supporting information

Supporting Information S1

## Data Availability

The data that support the findings of this study are available from the corresponding author upon reasonable request.
