# Supplementary material for: Sputum Eosinophil and Macrophage Changes After Aspirin Challenge in Patients With Nonsteroidal Anti‐Inflammatory Drug–Exacerbated Respiratory Disease
Source: Clin Transl Allergy. 2025 Sep 10;15(9):e70079. doi: 10.1002/clt2.70079 (PMC12421734; doi:10.1002/clt2.70079)
Supplement: Supplementary file 1 — Supporting Information S1 [file CLT2-15-e70079-s001.docx]

**Supplementary Materials**

**Sputum eosinophil and macrophage changes after aspirin challenge in patients with**

**nonsteroidal anti-inflammatory drug–exacerbated respiratory disease**

**Short title: Sputum eosinophils and macrophages after aspirin challenge in N-ERD**

Gabriela Trąd-Wójcik^1,2^, Piotr Szatkowski^1^, Adam Ćmiel^3^, Radosław Kacorzyk^1,2^, Adam Stępień^2^, Lucyna Mastalerz^1^

^1^ 2nd Department of Internal Medicine, Jagiellonian University Medical College, Krakow,

Poland

^2^ Doctoral School of Medical and Health Sciences, Jagiellonian University, Krakow, Poland

^3^ Department of Applied Mathematics, AGH University of Science and Technology, Krakow,

Poland

**Address for correspondence:**

2nd Department of Internal Medicine, Jagiellonian University Medical

Jakubowskiego 2, 30-688 Krakow, Poland

email: lucyna.mastalerz@uj.edu.pl

**Statistical analysis**

Descriptive statistics for demographic, clinical, and laboratory characteristics were presented as median with 25^th^ and 75^th^ percentiles for continuous variables and frequencies with percentages for categorical variables. The general linear model was used for group comparisons also taking into account repeated measure factor (with 2 levels before and after provocation). Logarithmic or Box-Cox transformations were used as variance-stabilizing transformation, when needed. When the transformations were unsuccessful, the nonparametric Kruskal-Wallis analysis of variance was used. The post-hoc Tukey test or the nonparametric Kruskal-Wallis test was used to control the family-wise error rate for multiple comparisons. Correlations between variables were assessed with the Spearman rank order correlation coefficient. Benjamini-Hochberg adjusted *p*-values were used to control the false discovery rate in the search for significant correlations. A *p*-value of 0.05 or lower was considered significant. Statistical analysis was performed with Statistica 13.3 (TIBCO Software Inc., Santa Clara, USA).

**Results**

**Characteristics of the study groups**

**Comparison of the N-ERD (n=52) and ATA (n=23) groups before oral aspirin challenge**

CRSwNPs was more common in the N-ERD than in the ATA group (100% vs. 91.3%, P=0.034). At baseline, mean FEV_1_ was lower in the N-ERD than in the ATA group (92.7±14.4 ml vs. 100.7±16.0 ml, P=0.036), while mean blood eosinophil count was higher in the N-ERD group (472.2±362.9/mm3 vs. 302.6±141.0/mm3, P=0.039). The median Lund-Mackay and SNOT-22 scores were higher in the N-ERD group than in the ATA group (15 [IQR, 13-2] vs. 14 [IQR, 11-14], P=0.036; 50 [IQR, 39-62] vs. 34 [IQR, 29-40], P=0.002, respectively). No differences were found in age, sex, BMI, asthma control and severity, ICS dose, asthma duration, IgE levels, and frequency of positive skin prick test results.

**Comparison of the N-ERD (n=26) and ATA (n=16) groups before inhaled aspirin challenge**

CRSwNPs was more common in the N-ERD than in the ATA group (100% vs. 68.8%, P=0.002). Mean blood eosinophil count was higher in the N-ERD than in the ATA group (467.8±272.9/mm^3^ vs. 210.5±176.1/mm^3^, P=0.001). The median Lund-Mackay and SNOT-22 scores were higher in the N-ERD than in the ATA group (18 [IQR, 13-21] vs. 7 [IQR, 3-10], P=0005; 36 [IQR, 29-41] vs. 20 [IQR, 15-23]; P=0.026, respectively). No differences were found in age, sex, BMI, asthma control and severity, baseline FEV_1_, ICS dose, asthma duration, IgE levels, and frequency of positive skin prick test results.

**Induced sputum cell counts**

**Sputum neutrophil percentage** **at baseline and after oral aspirin challenge in the N-ERD (n=52) and ATA (n=23)**

At baseline, there were no differences in sputum neutrophil percentage between groups. In addition, no significant changes were noted after the oral aspirin challenge in either group (Figure 2B in the main manuscript file).

**Sputum neutrophil percentage** **at baseline and after inhaled aspirin challenge in the N-ERD (n=26) and ATA (n=16) groups**

There were no differences in sputum neutrophil percentage between groups at baseline or after the inhaled aspirin challenge in either group (Figure 2B in the main manuscript file).

**Sputum lymphocyte percentage** **at baseline and after oral aspirin challenge in the N-ERD (n=52) and ATA (n=23) groups**

At baseline, there were no differences in sputum lymphocyte percentage between groups. In addition, no significant changes were noted after the oral aspirin challenge (Figure 2C in the main manuscript file).

**Sputum lymphocyte percentage** **at baseline and after inhaled aspirin challenge in the N-ERD (n=26) and ATA (n=16) groups**

There were no differences in sputum lymphocyte percentage at baseline or after inhaled aspirin challenge in either group (Figure 2C in the main manuscript file).

**Blood eosinophil count before and after oral aspirin challenge**

In the N-ERD group, blood eosinophil count decreased after oral aspirin challenge (6.49%±4.55% vs 4.64%±3.76%; P=0.005). No differences were observed in the ATA group.
